# Psychological safety and Chinese PreK-12 teachers organizational citizenship behavior: the mediating role of interpersonal trust

**DOI:** 10.3389/fpsyg.2026.1786530

**Published:** 2026-03-19

**Authors:** Xingping Zhou, Baoan Feng

**Affiliations:** College of Teacher Education, Quzhou University, Zhejiang, China

**Keywords:** Chinese PreK-12 teachers, interpersonal trust, mediation analysis, organizational citizenship behavior, psychological safety

## Abstract

**Introduction:**

While the positive association between psychological safety and organizational citizenship behavior has been well established, the mediating mechanisms underlying this relationship remain insufficiently explored, especially in the context of Chinese PreK-12 education. This study aims to examine the potential mediating role of interpersonal trust in explaining how psychological safety relates to organizational citizenship behavior among PreK-12 teachers in China.

**Methods:**

Data were collected from 620 PreK-12 teachers across China through an anonymous online survey. Participants provided their demographic information and completed validated scales measuring psychological safety, interpersonal trust, and organizational citizenship behavior. The survey data were then analyzed to test the proposed mediating effect of interpersonal trust.

**Results:**

The results indicated that both psychological safety and interpersonal trust were positively correlated with organizational citizenship behavior. Further mediating effect analysis revealed that interpersonal trust played a partial mediating role in the relationship between psychological safety and organizational citizenship behavior. Specifically, psychological safety promotes organizational citizenship behavior in part by enhancing teachers' interpersonal trust.

**Discussion:**

These findings provide a clear conceptual pathway for understanding how to foster organizational citizenship behavior among Chinese PreK-12 teachers. The study discusses the theoretical contributions of revealing the partial mediating role of interpersonal trust, the practical implications for improving teachers' organizational citizenship behavior in PreK-12 educational settings, and the limitations of the current research.

## Introduction

In the global context of educational quality improvement, teachers' voluntary, role-transcending behaviors—conceptualized as organizational citizenship behavior—are increasingly recognized as critical drivers of school effectiveness and student development (Phetsombat and Na-Nan, 2023; Sun H. et al., 2025). As a core construct in organizational behavior, organizational citizenship behavior refers to employees' discretionary actions that exceed formal job requirements and contribute to organizational wellbeing (Organ, 1988). For teachers, such behaviors (e.g., taking the initiative to assist colleagues in resolving teaching problems and jointly addressing unfinished administrative affairs) can enhance both individual teachers' personal efficacy and the overall efficacy of the team (Erturk, 2015). Research indicates that teacher organizational citizenship behavior supports the cultivation of positive attitudes and behaviors in students, strengthens team cohesion among educators, and ultimately contributes to the overall success of schools (Phetsombat and Na-Nan, 2023).

Given the significant value of teacher organizational citizenship behavior, identifying and understanding its antecedents has been a persistent focus in organizational behavior research. Prior studies have established that organizational-level factors are important predictors of individual organizational citizenship behavior, including leadership styles (e.g., ethical leadership; Phetsombat and Na-Nan, 2023), learning culture (Idris et al., 2021), organizational democracy (Haskasap et al., 2023), organizational justice (Rahman and Karim, 2022), and organizational support and trust (Im and Chung, 2018). Notably, while these organizational factors provide a structural foundation for teacher organizational citizenship behavior, their effectiveness in motivating such discretionary behaviors often hinges on a critical psychological mechanism: psychological safety. Defined as a shared belief that team members can speak up, take risks, and admit mistakes without fear of negative consequences (Edmondson, 1999), psychological safety mitigates employees' defensive concerns and fosters proactive engagement—two prerequisites for organizational citizenship behavior. Consistently, empirical research has confirmed that psychological safety is positively associated with higher levels of organizational citizenship behavior among individuals (e.g., Iqbal et al., 2022; Liu and Keller, 2021). This is because psychologically safe environments help overcome the fear of negative consequences, reduce defensive behaviors, and encourage proactive contributions (Feng et al., 2025; Iqbal et al., 2022; Mogård et al., 2023).

Despite these advancements, it remains unclear through which specific pathways psychological safety exerts its influence on individual organizational citizenship behavior, and whether additional variables mediate this relationship. One plausible mechanism is interpersonal trust. Previous studies have primarily explored how psychological safety impacts organizational citizenship behavior from the perspective of positive psychology (Mansour and Tremblay, 2018; Yang et al., 2023), yet the underlying mechanism has not been analyzed from the lens of cognitive and behavioral tendencies in social interactions—such as interpersonal trust. In fact, psychological safety has been shown to positively facilitate social interactions (Jowett et al., 2023). At the social interaction level, factors such as social network structure (Tsang et al., 2012), coworkers' friendship behaviors (Ollier-Malaterre and Foucreault, 2021), and team cohesion (Cantisano and Domínguez, 2006) further exert positive influences on individual organizational citizenship behavior through group dynamics and reciprocal norms. As a core component of social interactions, interpersonal trust serves as a “fundamental support” in daily communication, team collaboration, and the maintenance of broader social relationships (Blau, 1964). Existing research also indicates a positive correlation between interpersonal trust and organizational citizenship behavior (Singh and Srivastava, 2009).

Furthermore, our review of relevant literature reveals that most studies have focused on the psychological safety, interpersonal trust, and organizational citizenship behavior of company employees. However, very little attention has been paid to school teachers, especially in the context of Chinese culture. As a typical collectivistic society, China prioritizes interpersonal harmony and reciprocal relationships—values that are particularly salient in the teaching profession, where collaborative practice is integral to daily work (He and Wei, 2022; Zhao et al., 2025). Against this cultural backdrop, exploring how psychological safety interacts with interpersonal trust to shape teachers' organizational citizenship behavior not only addresses existing theoretical gaps but also provides context-specific insights for educational management. Based on the above analysis, this study takes interpersonal trust as a mediating variable to systematically elaborate on the mechanism underlying the impact of psychological safety on organizational citizenship behavior, thereby providing theoretical support and practical implications for enhancing organizational citizenship behavior among Chinese PreK-12 teachers.

## Theoretical background and hypotheses

### Social exchange theory

Social exchange theory provides the foundational explanatory framework for this study. This theory posits that human relationships are built upon a series of implicit reciprocal exchanges, where individual actions are driven by the expectation of future valuable returns (Cropanzano and Mitchell, 2005). Such exchanges extend beyond economic or material resources to encompass socioemotional resources like affective support, social recognition, respect, and trust (Blau, 1964). When individuals perceive goodwill, investment, or support from their organization or colleagues, they develop an obligation to reciprocate. This sense of indebtedness makes them more likely to exhibit positive behaviors that exceed formal role requirements, thereby maintaining balance and mutuality in the exchange relationship (Gouldner, 1960).

In the context of this research, psychological safety serves as the critical situational condition that initiates and sustains high-quality social exchange. A school environment characterized by high psychological safety allows teachers to freely express opinions, raise questions, or admit mistakes without fear of negative evaluation or punishment (Edmondson, 1999). Such an environment represents a significant form of collective socioemotional investment from the organization and the peer group, fostering a shared perception of relational security.

According to the logic of social exchange, teachers who perceive this collective investment are motivated to reciprocate. However, the translation of this psychological connection to the environment into sustained extra-role behavior requires a crucial intermediary mechanism: the development of robust interpersonal trust. While psychological safety establishes a baseline of relational security at the team or organizational level (“it is safe to speak up here”), interpersonal trust operationalizes this security into dyadic or small-group expectations about specific others (“I can rely on my colleagues/leaders”).

The transition from individual-level trust to a team-level climate of trust is pivotal. Initially, psychological safety facilitates risk-taking in interpersonal interactions, allowing trust to germinate between individuals (Carmeli et al., 2009). As these dyadic trust ties multiply and interconnect through repeated positive exchanges, they coalesce into a shared perception of interpersonal trust at the team level—a generalized belief in the reliability, integrity, and benevolence of fellow team members (Fulmer and Gelfand, 2012). This team-level trust climate significantly reduces social uncertainty and transaction costs associated with cooperative actions (Rousseau et al., 1998).

Thus, within the social exchange framework, interpersonal trust—particularly when it escalates to a shared team property—functions as the central bond that sustains exchange relationships (Blau, 1964). It transforms the diffuse sense of obligation arising from psychological safety into a confident expectation that one's extra-role contributions will be recognized, valued, and potentially reciprocated by the collective in the long run (Konovsky and Pugh, 1994). In summary, this study proposes a mediation mechanism grounded in social exchange theory: a psychologically safe environment acts as an initial collective investment. This investment nurtures interpersonal trust, which evolves from individual assessments into a team-level relational asset. This asset, in turn, empowers teachers to confidently engage in organizational citizenship behaviors, thereby fulfilling their reciprocal obligations and reinforcing the cycle of high-quality social exchange.

### Psychological safety and organizational citizenship behavior

In school settings, psychological safety generates profound positive effects on teachers' professional wellbeing and organizational functionality. It acts as a pivotal precursor to desirable workplace outcomes, such as mitigated teacher burnout, strengthened professional self-efficacy, and optimized perceptions of the school's organizational climate (Fleming et al., 2024). By reducing the fear of negative evaluation and interpersonal friction, a psychologically safe environment nurtures collaborative interactions among teachers, fostering a supportive school culture that enhances collective learning and team effectiveness (Newman et al., 2016). This enabling context further motivates teachers to engage in work-related exchanges beyond formal job obligations (Frazier et al., 2017). Notably, psychological safety is closely intertwined with teachers' organizational citizenship behavior (Iqbal et al., 2022; Liu and Keller, 2021). Grounded in social exchange theory, psychological safety cultivates a sense of mutual trust and support, prompting teachers to reciprocate the favorable organizational context through voluntary positive behaviors (Newman et al., 2016). When teachers feel psychologically safe, they are more inclined to partake in activities benefiting colleagues and the school, such as sharing teaching resources, assisting peers with work difficulties, and contributing to school improvement efforts (Agarwal and Anantatmula, 2023). Empirical studies have corroborated that psychological safety positively predicts organizational citizenship behavior by fostering a proactive work climate where individuals feel secure to act without fear of negative consequences (Din et al., 2024). Based on the above reasoning, the following hypothesis is proposed:

*Hypothesis 1: Psychological safety is positively correlated with organizational citizenship behavior among Chinese PreK-12 teachers*.

### Interpersonal trust as a mediator

Interpersonal trust, within organizational contexts, is defined as an individual's willingness to accept vulnerability based on the positive expectations that other parties will perform actions beneficial to one's interests or not engage in behaviors harmful to one's interests (Edmondson, 2019; Rousseau et al., 1998). It is a relational construct central to social exchange, facilitating cooperative actions by reducing uncertainty and perceived risk in interactions among organizational members (Fulmer and Gelfand, 2012). The establishment and reinforcement of interpersonal trust are significantly influenced by the climate of psychological safety. A psychologically safe environment, characterized by mutual respect and non-punitive responses to interpersonal risk-taking, fosters conditions conducive to trust development (Baeva and Bordovskaia, 2015). When teachers feel secure in voicing concerns or admitting mistakes without fear of embarrassment or retribution, it signals reliability and benevolent intent from colleagues and leaders, thereby strengthening positive expectations about others' behaviors (Gonda et al., 2024; Xie et al., 2022). This process aligns with the tenets of social exchange theory (Blau, 1964), where perceptions of safety encourage reciprocal, trust-building exchanges. Empirical research supports this linkage, indicating that psychological safety is a robust antecedent to trust, as it lowers the defensive mechanisms that typically inhibit open and trusting relationships (Roussin and Webber, 2012; Walker, 2025). Subsequently, heightened trust acts as a critical catalyst for organizational citizenship behavior (Kim and Park, 2025; Oh, 2022). Trust can positively foster organizational identification among individuals, and such identification in turn prompts them to display more organizational citizenship behavior that benefits the organization and colleagues (Kim, 2019). Studies in educational settings further corroborate that trust among teachers is positively associated with increased collaboration and voluntary, citizenship-oriented contributions (Choong and Ng, 2023, 2024). Therefore, based on the reasoned pathway from climate to relational states to behavioral outcomes, the following hypothesis is proposed:

*Hypothesis 2: Interpersonal trust mediates the positive relationship between psychological safety and organizational citizenship behavior among Chinese PreK-12 teachers*.

**Figure 1:**
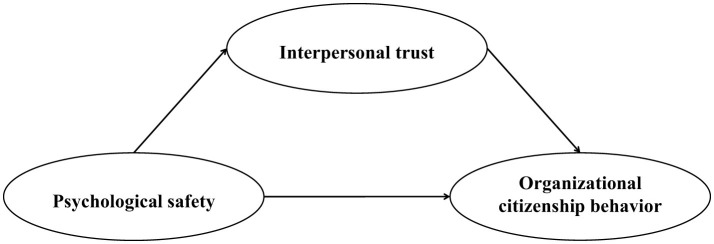
The hypothesized model.

Rooted in social exchange theory, this study advances a conceptual framework that delineates interpersonal trust as a mediating variable accounting for the relationship between psychological safety and organizational citizenship behavior among PreK-12 teachers in China (see Figure 1).

## Methods

### Study design and participants

This study adopted a cross-sectional design, aiming to explore the potential mediating role of interpersonal trust between psychological safety and organizational citizenship behavior. The research subjects were kindergarten, primary school, and secondary school teachers in China. The research protocol (approval number: removed for peer review) was approved by the ethics review committee on January 13, 2025, in accordance with the principles of the Helsinki Declaration. All data obtained in this study were stored on an encrypted computer maintained by the project leader to ensure security and prevent unauthorized access.

The data collection for this cross-sectional survey was conducted from November 13 to November 29, 2025, using a convenience sampling method to recruit Chinese PreK-12 teachers (including pre-kindergarten, primary school, and secondary school teachers). This process involved the following steps: firstly, an electronic questionnaire was compiled on the Wjx platform (www.wjx.cn). The questionnaire included an informed consent statement, detailed instructions for filling out, and standardized scales for assessing psychological safety, interpersonal trust, and organizational citizenship behavior, followed by a section on demographic information (covering age, gender, marital status, tenure, educational background, and teaching stage). Secondly, the questionnaire link was distributed to familiar target contacts through WeChat, QQ, and DingTalk (the most popular APP for both office and social functions among Chinese teachers), who were distributed across different regions in China, and they were entrusted to help forward the questionnaire link to the work groups (or platforms) of their respective institutions to recruit more voluntary participants. Finally, before formally filling out the electronic questionnaire, each participant had to carefully read the informed consent form and click “agree to participate” to be considered as having given informed consent, after which they could continue to answer the survey questions. Inclusion criteria were: (i) teachers currently employed in a formal school (including pre-kindergarten); (ii) required to be in full-time employment and have at least 6 months of teaching experience; (iii) officially signed labor contracts. Exclusion criteria were: (i) tutors working in non-full-time community institutions; (ii) teachers who have been on leave or on vacation for more than 3 months; (iii) university teachers, adult education teachers, intern teachers, or substitute teachers. A total of 650 respondents were initially recruited through online questionnaires, and 30 responses were excluded due to abnormal response patterns (such as linear responses) or unreasonable completion times (less than 5 min or more than 15 min). After excluding these data, this study retained 620 valid responses for analysis, with a final retention rate of 95.38%.

A priori sample size estimation was conducted using G^*^Power (Faul et al., 2009). With a medium effect size (*f*^2^ = 0.10), significance level (α) of 0.05, statistical power of 0.95, and accounting for eight predictor variables, the analysis established a minimum of 236 participants. This target was greatly exceeded by the final sample of 620, which bolsters the reliability and generalizability of the study's conclusions.

### Measures

#### Psychological safety

Psychological safety was measured with an eight-item scale adapted from the Team Climate Inventory (Anderson and West, 1996) by (Yu et al. 2016). This adapted scale focuses on core dimensions of psychological safety including interpersonal openness, mutual support, information sharing, safe expression of ideas, and acceptance among team members, which directly reflect the core characteristics of psychological safety (Edmondson, 1999). The selected items emphasize the extent to which teachers feel safe, supported, and accepted to express opinions and communicate openly within the team. All items were rated on a five-point Likert scale (1 = *strongly disagree*, 5 = *strongly agree*); higher total scores indicate greater perceived psychological safety (e.g., “In a team, immature viewpoints can also be valued”). The reliability and validity of this Chinese version for measuring teacher psychological safety have been well supported in prior educational studies (e.g., Miao et al., 2020). In the current sample, the scale showed excellent internal consistency, with Cronbach's α = 0.956.

#### Interpersonal trust

Interpersonal trust was measured using a seven-item Chinese adaptation (Lu et al., 2006) of the Interpersonal Trust at Work scale (Cook and Wall, 1980). The scale is theoretically two-dimensional, assessing trust in others' intentions and confidence in actions. The assessment targets of this scale include both peers and superiors. We followed the research design of (Lu et al. 2006) and focused our assessment solely on the relationship with peers. Responses were recorded on a seven-point Likert scale (1 = *strongly disagree*, 7 = *strongly agree*), with higher total scores indicating greater perceived interpersonal trust toward colleagues (e.g., “Overall, I can trust my colleagues to do what they say”). The Chinese version has established satisfactory reliability in prior research (Lu et al., 2006). In the present study, the scale exhibited high internal consistency (Cronbach's α = 0.919).

#### Organizational citizenship behavior

Teachers' organizational citizenship behavior was evaluated using the 10-item scale developed by (Bachrach et al. 2007). The scale comprises two subdimensions: helping behavior (items 1–7) and civic virtue (items 8–10). A sample item reads, “I take the initiative to spare time to help colleagues who encounter work difficulties.” Responses were provided on a seven-point Likert scale (1 = *strongly disagree*, 7 = *strongly agree*), with higher scores reflecting stronger engagement in organizational citizenship behavior. A previous study using Chinese samples has reported satisfactory reliability for this instrument (Zhu and Yang, 2025). In the present study, the full scale demonstrated excellent internal consistency, with Cronbach's α = 0.933.

Notably, different Likert response scales (5-point for psychological safety, 7-point for interpersonal trust and organizational citizenship behavior) were retained to preserve the original, validated response formats of each established instrument, consistent with prior validation research. Standard statistical procedures are robust to such scale differences and do not affect the interpretation of structural relations.

#### Control variables

In the present study, age, gender, tenure, marital status, and educational attainment were incorporated as control variables to account for their potential confounding effects on the core variables. Existing literature indicates that these demographic factors are associated with the key constructs under investigation, yet the findings remain inconsistent. Regarding psychological safety, some studies have documented positive correlations with age, gender, and marital status (Feng et al., 2025), while educational attainment may exhibit a negative association (Wang et al., 2024). For interpersonal trust, evidence suggests that age may negatively predict trust levels, and males tend to demonstrate lower trust propensity compared to females (Wang et al., 2022). Concerning organizational citizenship behavior, most research has shown that females exhibit higher levels of such behavior (Bayati et al., 2025; Khusanova et al., 2019); age, tenure, and educational background are generally positively correlated with it (Ha and Moon, 2023; Jang, 2021), and married employees may also display elevated organizational citizenship behavior (Sun J.-X. et al., 2025). Given these inconsistent findings, the current study controlled for the aforementioned variables in statistical analyses to more clearly delineate the core relationships among psychological safety, interpersonal trust, and organizational citizenship behavior.

### Statistical analysis

There were no missing data across the 620 valid samples included in the final analysis. Data analysis was performed using SPSS 29.0 and Amos 29.0. Descriptive statistics were first computed for all demographic variables. Pearson correlation analysis was then conducted to examine the relationships among the demographic variables, psychological safety, interpersonal trust, and organizational citizenship behavior. Subsequently, confirmatory factor analysis (CFA) was carried out in Amos 29.0 to assess common method bias and evaluate the discriminant validity of the measurement model. The discriminant validity was examined by comparing the fit indices of three competing models. Model fit was evaluated using several widely accepted indices, including the chi-square statistic (χ^2^), Comparative Fit Index (CFI), Tucker–Lewis Index (TLI), Goodness-of-Fit Index (GFI), and Root Mean Square Error of Approximation (RMSEA). To test the hypothesized mediation model in which interpersonal trust mediates the relationship between psychological safety and organizational citizenship behavior, Model 4 of the PROCESS macro for SPSS (version 4.1) was applied (Hayes, 2018). Model 4 was selected because it is specifically designed for single-mediator models, allows simultaneous estimation of direct, indirect, and total effects, and provides robust statistical inference via bias-corrected bootstrapping, which is the most recommended method for examining mediation effects. The significance of the indirect effect was examined using a bias-corrected bootstrapping procedure with 5,000 resamples. An indirect effect was considered statistically significant if its 95% corrected confidence interval (CI) did not include zero (Hayes, 2018).

## Results

### Descriptive statistics

The sample comprised 620 teachers. Their mean (M) age was 33.39 years, with a standard deviation (SD) of 9.29, ranging from 18 to 60, with an average organizational tenure of 11.18 years (SD = 10.07). The sample was predominantly female (82.58%) and married (62.90%). Regarding educational attainment, the vast majority held a bachelor's degree (87.10%), with a small portion having an associate degree (7.42%) or graduate education (5.48%). In terms of educational stage, the teachers were distributed as primary school (41.45%), preschool (36.77%), and secondary school (21.77%) educators. The values of mean and standard deviation corresponding to psychological safety, interpersonal trust, and organizational citizenship behavior were 4.20 (0.74), 5.66 (1.14), and 5.88 (1.11) in sequence (see Table 1).

**Table 1:** Coding structure and distributional characteristics of all variables (*n* = 620).

**Variable**	**Category/Statistic**	***n*(*%*)/Mean (SD)**
Age	Range 18–60	33.39 (SD = 9.29)
Gender	0 = Male	108 (17.42)
	1 = Female	512 (82.58)
Marital status	0 = Unmarried	230 (37.10)
	1 = Married	390 (62.90)
Tenure	Range 1–46	11.18 (SD = 10.07)
Educational attainment	1 = Associate degree	46 (7.42)
	2 = Bachelor's degree	540 (87.10)
	3 = Graduate education	34 (5.48)
Educational stage	1 = Preschool teachers	228 (36.77)
	2 = Primary school teachers	257 (41.45)
	3 = Secondary school teachers	135 (21.77)
Psychological safety	Total sample	4.20 (0.74)
Interpersonal trust	Total sample	5.66 (1.14)
Organizational citizenship behavior	Total sample	5.88 (1.11)

### Measurement model

To assess potential common method bias, a single-factor confirmatory factor analysis was conducted within the structural equation modeling framework, following the widely adopted procedure proposed by (Harris and Mossholder 1996). Using Amos 29.0, all items of the key variables were specified to load onto a single common factor. The results demonstrated extremely poor model fit (χ^2^/df = 18.136, CFI = 0.703, TLI = 0.676, RMSEA = 0.166), indicating that a single common method factor could not adequately account for the variance among the measures. Accordingly, common method bias was not a significant concern in the present study. Subsequently, to ensure the validity of the regression results, multicollinearity was assessed prior to the main analysis. The variance inflation factor (VIF = 2.455) and tolerance (Tolerance = 0.407) statistics for all independent variables met the widely accepted diagnostic thresholds (VIF < 10 and Tolerance > 0.10; e.g., Hair et al., 2019), indicating that multicollinearity does not pose a substantial threat to the parameter estimates.

A series of confirmatory factor analyses (Amos 29.0) comparing theoretically-derived measurement structures identified the three-factor model as optimally representing the data (see Table 2). Its fit indices (χ^2^/*df* = 2.660, CFI = 0.975, TLI = 0.969, GFI = 0.925, RMSEA = 0.052) met or exceeded standard cut-offs (Ferreira-Valente et al., 2016), empirically validating the discriminant validity and structural precision of the three-dimensional construct.

**Table 2:** Comparison of fit indices for latent structural equation models.

**Model**	***χ^2^***	***χ^2^/df***	***CFI***	***TLI***	***GFI***	***RMSEA***
Model 1	4,987.383	18.136	0.703	0.676	0.472	0.166
Model 2	3,099.330	11.311	0.822	0.805	0.637	0.129
Model 3	630.380	2.660	0.975	0.969	0.925	0.052

Model 1: All items of psychological safety, interpersonal trust, and organizational citizenship behavior were incorporated into a single latent factor. Model 2: Psychological safety and interpersonal trust were integrated into a single comprehensive latent factor, while organizational citizenship behavior was treated as an independent latent factor. Model 3: Psychological safety, interpersonal trust, and organizational citizenship behavior were each specified as three mutually independent latent factors.

As shown in Table 3, the Cronbach's alpha and composite reliability (CR) values for all key constructs (psychological safety, interpersonal trust, and organizational citizenship behavior) exceeded 0.90, indicating excellent internal consistency. The average variance extracted (AVE) for each construct was greater than 0.50 (Ferreira-Valente et al., 2016), supporting convergent validity. Furthermore, the square root of each construct's AVE (shown in bold on the diagonal) was greater than its correlations with other constructs (Fornell and Larcker, 1981), providing evidence of discriminant validity.

**Table 3:** Descriptive statistics, reliability and validity indices, and correlations (*n* = 620).

**Variables**	**α**	**AVE**	**CR**	**1**	**2**	**3**	**4**	**5**	**6**	**7**	**8**
1. Age				1							
2. Gender				−0.205^***^	1						
3. Tenure				0.939^***^	−0.222^***^						
4. Marital status				0.632^***^	−0.062	0.575^***^					
5. Educational attainment				−0.10^*^	−0.037	−0.114^**^	−0.023				
6. Psychological safety	0.956	0.733	0.956	0.148^***^	−0.002	0.154^***^	0.164^***^	−0.125^**^	**0.856**		
7. Interpersonal trust	0.919	0.645	0.926	0.168^***^	−0.029	0.183^***^	0.182^***^	−0.118^**^	0.770^***^	**0.803**	
8. Organizational citizenship behavior	0.933	0.639	0.945	0.234^***^	0.015	0.240^***^	0.232^***^	−0.077	0.688^***^	0.746^***^	**0.799**
M (SD)				33.39 ± 9.29	0.83 ± 0.38	11.18 ± 10.07	0.63 ± 0.48	1.98 ± 0.36	4.20 ± 0.74	5.66 ± 1.14	5.88 ± 1.11

Diagonal elements (the bolded values) show the square roots of average variance extracted (AVE) for assessing discriminant validity. ^*^p < 0.05, ^**^p < 0.01, and ^***^p < 0.001.

### Correlation analysis

Table 3 presents the descriptive statistics and the correlation matrix. Correlation analysis revealed that the three core variables were all significantly and positively intercorrelated (*r* values ranged from 0.69 to 0.77, all *p* < 0.001). These results offer preliminary empirical evidence for the subsequent structural model testing.

### Hypotheses testing

The analysis examined the proposed mediation model using PROCESS macro Model 4 (Hayes, 2018), after controlling for age, gender, tenure, marital status, and educational attainment. As presented in Table 4, the regression model for interpersonal trust accounted for 59.9% of its variance, with psychological safety showing a strong positive association [β = 1.165, *p* < 0.001, 95% CI (1.086, 1.244)]. None of the demographic covariates were significantly related to interpersonal trust. For organizational citizenship behavior, the full model explained 60.5% of the variance. When both psychological safety and interpersonal trust were included as concurrent predictors, both demonstrated significant positive associations with organizational citizenship behavior [psychological safety: β = 0.410, *p* < 0.001, 95% CI (0.292, 0.528); interpersonal trust: β = 0.503, *p* < 0.001, 95% CI (0.427, 0.580)]. Among the control variables, only gender was uniquely associated with organizational citizenship behavior [β = 0.163, *p* < 0.05, 95% CI (0.012, 0.314)]. The above findings support Hypothesis 1.

**Table 4:** Summary of effects in the mediation model.

**Predictors**	Interpersonal trust			Organizational citizenship behavior		
	β	*SE*	*95% CI*	β	*SE*	*95% CI*
Age	−0.012	0.010	[−0.031, 0.007]	0.006	0.009	[−0.012, 0.025]
Gender	−0.051	0.080	[−0.208, 0.105]	0.163^*^	0.077	[0.012, 0.314]
Tenure	0.014	0.009	[−0.003, 0.031]	0.006	0.008	[−0.011, 0.022]
Marital status	0.108	0.079	[−0.048, 0.263]	0.083	0.077	[−0.067, 0.233]
Educational attainment	−0.058	0.083	[−0.221, 0.104]	0.099	0.080	[−0.059, 0.256]
Psychological safety	1.165^***^	0.040	[1.086, 1.244]	0.410^***^	0.060	[0.292, 0.528]
Interpersonal trust				0.503^***^	0.039	[0.427, 0.580]
*R^2^*	0.599			0.605		
*F*	152.614^***^			133.784^***^		

^*^p < 0.05; ^***^p < 0.001. SE, Standard Error. The analysis employed 5,000 bootstrap iterations to ensure robust parameter estimation.

**Figure 2:**
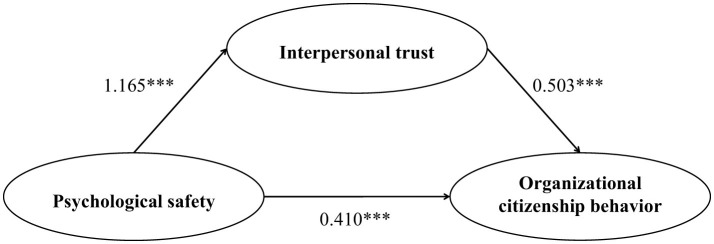
Path coefficients for the mediated relationship of psychological safety with organizational citizenship behavior through interpersonal trust. ***p < 0.001.

The indirect association was tested via a bootstrap estimation with 5,000 iterations. The product of the coefficient from psychological safety to interpersonal trust and the coefficient from interpersonal trust to organizational citizenship behavior yielded an indirect effect estimate of 0.586 [95% CI (0.383, 0.752)]. The bootstrap-derived 95% bias-corrected confidence interval for this indirect effect did not include zero, indicating a statistically significant indirect association. The direct association between psychological safety and organizational citizenship behavior, while remaining significant, was reduced in magnitude (β = 0.410) compared to its total association (β = 0.996) prior to entering interpersonal trust into the model. The indirect association accounted for approximately 58.8% of the total observed association between psychological safety and organizational citizenship behavior. The remaining significant direct effect suggests that psychological safety can also promote organizational citizenship behavior through other plausible mechanisms beyond interpersonal trust, such as enhanced work engagement (Mansour and Tremblay, 2018). In summary, the results are consistent with the hypothesized mediating role of interpersonal trust (Hypothesis 2 is supported). Bootstrap analysis confirmed the significance of the indirect pathway. The pattern of associations suggests that the positive relationship between psychological safety and organizational citizenship behavior can be partially understood through its shared relationship with interpersonal trust (Figure 2).

## Discussion

This study examined the mediating role of interpersonal trust in the relationship between psychological safety and organizational citizenship behavior among Chinese PreK-12 teachers. The findings support the proposed hypotheses, revealing a significant positive association between psychological safety and organizational citizenship behavior, and further identifying interpersonal trust as a significant partial mediator. This discussion interprets these results, elucidates their theoretical contributions, and outlines practical implications while acknowledging the study's limitations.

Consistent with previous research in various organizational contexts (Din et al., 2024; Frazier et al., 2017), our results confirm that psychological safety is positively correlated with teachers' organizational citizenship behavior. In the collectivist environment of Chinese schools, where hierarchical norms can sometimes inhibit voice (Zhao et al., 2025), a climate where teachers feel safe to take interpersonal risks, voice concerns, and admit mistakes without fear of negative consequences appears to be a powerful catalyst for discretionary, pro-organizational actions. This finding reinforces the universality of the psychological safety—organizational citizenship behavior link while highlighting its particular relevance in educational systems undergoing reform and demanding high adaptability from educators.

More importantly, this study extends the literature by unpacking the “black box” of this relationship. The significant mediating role of interpersonal trust suggests that psychological security is not only directly related to teachers' organizational citizenship behavior, but also indirectly linked through the establishment of a solid trust foundation among colleagues and between teachers and school leaders. According to social exchange theory (Blau, 1964), when teachers perceive a psychologically safe environment, they interpret it as a form of organizational support and benevolent intent. This perception cultivates interpersonal trust—a belief in the reliability, integrity, and benevolence of others within the school (Tschannen-Moran and Hoy, 2000). This trust, in turn, establishes a normative framework of reciprocal obligations. Teachers who trust their peers and leaders are more likely to engage in extra-role behaviors such as helping colleagues, volunteering for additional duties, and proactively defending the school's reputation, as they believe their efforts will be recognized and reciprocated in the long term, rather than exploited (Dirks and Ferrin, 2002). Our findings also resonate with the contextual logic of collectivist cultures such as China, where interpersonal trust is widely regarded as a core social bond that facilitates the translation of positive organizational climate perceptions (e.g., psychological safety) into employees' cooperative and prosocial behaviors (Macy and Sato, 2002). In collectivist societies that emphasize interdependence and group harmony (He and Wei, 2022), the trust derived from a psychologically safe environment strengthens teachers' sense of organizational identification to the school community (Edmondson, 2019; Jurek et al., 2025), thereby motivating them to engage in more organizational citizenship behavior that benefits the collective rather than just individual interests (Sheikhi and Yousefi, 2023).

The present findings further reveal that interpersonal trust exhibits a relatively stronger direct association with organizational citizenship behavior than psychological safety does, indicating that interpersonal trust acts as a more proximal and direct driver of teachers' voluntary cooperative and prosocial behaviors. From a practical lens, this pattern implies that efforts to promote teachers' organizational citizenship behavior may gain tangible benefits from prioritizing strategies that enhance mutual trust among colleagues, such as facilitating collaborative work tasks, encouraging open and supportive interpersonal communication, and nurturing reciprocal help in daily work. Notably, psychological safety remains an indispensable foundational and climate-level prerequisite for this process: it is the psychologically safe organizational climate that creates the enabling context for the formation and sustenance of high levels of interpersonal trust between teachers, highlighting the complementary and hierarchical roles of psychological safety and interpersonal trust in shaping teachers' organizational citizenship behavior.

This partial nature of the mediation suggests that mechanisms beyond interpersonal trust may also be at play. However, our review of the existing literature did not identify clear, direct empirical evidence pointing to other specific pathways. Future research could build upon the present study by further investigating these potential alternative or complementary mechanisms. For instance, exploring factors such as organizational democracy (Haskasap et al., 2023), organizational justice (Rahman and Karim, 2022), or perceived organizational support (Im and Chung, 2018) as additional mediators or moderators would help to construct a more comprehensive theoretical model explaining how psychological safety influences organizational citizenship behavior in educational contexts.

### Theoretical contributions

This research makes two theoretical contributions. Firstly, our research has expanded the application scope and applicable conditions of social exchange theory (Blau, 1964). The consensus among scholars in this field holds that individuals, upon evaluating the anticipated benefits and costs of an interaction, proceed to engage in social exchange, a process fundamentally governed by the norm of reciprocity (Yeh and Huang, 2025). (Cropanzano and Mitchell 2005) contended that while the social exchange theory framework holds practical utility, its structural dimensions have yet to be fully established. This study provides modest but meaningful empirical evidence to inform the application and extension of social exchange theory in the context of educational settings, with a focus on interpersonal exchange dynamics among teachers. The research results indicate that the core principle of this theory—the principle of reciprocity—effectively explains the interpersonal behavioral dynamics in the context of Chinese school collectivism and hierarchy. Teachers' perception of a psychologically safe environment appears to be interpreted as an intangible, socio-emotional benefit from the organization. In order to continuously gain benefits, based on the basic norms of reciprocity, teachers will fulfill the obligations they should undertake after receiving benefits from the organization, that is, by performing organizational citizenship behaviors to repay the organization (Blau, 1964; Lian and Ding, 2025). Meanwhile, interpersonal trust establishes a more concrete normative framework for social exchange, making teachers' participation in organizational citizenship behavior a more likely reciprocal response. The application of this mechanism further strengthens the explanatory power and applicability of social exchange theory in explaining how “macro-level environmental perceptions are translated into specific relational and behavioral outcomes within educational organizations.”

Second, this study responds directly to the call in organizational behavior research for deeper investigations into psychological mechanisms within non-Western settings (Tsui, 2007). Many classical organizational behavior theories (e.g., social exchange theory) have been predominantly developed and validated in Western individualistic cultures (Hofstede, 2001), their explanatory pathways in collectivist, high-power-distance contexts like China's education system require further scholarly attention and contextual refinement. The core contribution of this study lies in specifying the culturally nuanced mediating mechanism of interpersonal trust between psychological safety and organizational citizenship behavior. Within the collective and hierarchical environment of Chinese PreK-12 schools, the function of psychological safety may diverge from Western conceptualizations. Rather than primarily enabling uninhibited individual voice, psychological safety in this context may operate by cultivating relationship security and affective bonds (Ok et al., 2025). Specifically, under Chinese cultural norms emphasizing guanxi (relationships) and harmony (Jia et al., 2023; Ma et al., 2023), a climate of safety is posited to foster affect-based trust—rooted in emotional ties and personal loyalty—particularly toward authority figures and among colleagues (McAllister, 1995). This form of trust, in turn, is theorized to be a pivotal socio-emotional driver prompting teachers to engage in extra-role behaviors that benefit the collective, such as voluntary collaboration and safeguarding the school's reputation (Chen et al., 2014). This proposed pathway challenges the more cognitively oriented trust mechanisms often highlighted in mainstream literature. Thus, the study moves beyond merely testing a Western-derived model. It illuminates a culturally specific process whereby psychological safety translates into organizational citizenship behavior through the central channel of relational and affect-laden trust. This insight reinforces the premise that the activation of employee behaviors is deeply embedded in cultural logics, contributing empirical evidence from the Chinese educational context toward building more inclusive and context-sensitive organizational theories.

### Implications for practice

The findings offer actionable insights for school administrators and educational policymakers in China and similar contexts. First, school leaders should proactively strive to create a psychologically safe organizational environment. Previous research has indicated a significant positive correlation between inclusive and democratic leadership styles and psychological safety (Imran et al., 2025; Yousaf et al., 2022). It is recommended that leaders adapt and adopt teacher-centered leadership styles, laying a solid foundation for fostering a psychologically safe environment. Meanwhile, providing professional training for all faculty and school leaders is also conducive to enhancing the level of psychological safety on campus (Porter-Stransky et al., 2024). Furthermore, we believe that creating psychological safety can be achieved by leaders modeling vulnerability (e.g., admitting their own mistakes), explicitly inviting input and dissenting opinions during meetings, responding to suggestions and concerns constructively rather than defensively, and framing failures as opportunities for learning rather than occasions for blame. Second, deliberately foster interpersonal trust. Since trust serves as a critical communication channel, policies and practices that enhance credibility should be prioritized. Given the empirical finding of a positive correlation between psychological safety and interpersonal trust, we recommend that schools: treat the cultivation of psychological safety as the starting point for systematic trust-building, implement transparency management (e.g., collective consultation mechanisms for important decisions), establish predictable supportive systems (e.g., non-punitive teaching reflection mechanisms), and organize collaborative activities that promote emotional connection (e.g., interdisciplinary teaching research and mentorship programs). These measures aim to translate organizational psychological safety into tangible, trust-enhancing behaviors, thereby providing institutional stability and interactive spaces for the sustained development of interpersonal trust. Third, integrated interventions. School improvement initiatives should consider an integrated approach that simultaneously targets psychological safety and trust to promote organizational citizenship behavior. For instance, mentoring programs for new teachers or cross-grade teacher collaboration projects should be designed not only for skill transfer but also with explicit norms that encourage open dialogue and mutual support, thereby strengthening both safety and trust. Fourth, performance appraisal and public recognition. While organizational citizenship behavior is by definition discretionary, school systems can recognize and appreciate such behaviors publicly. Recognizing acts of collaboration and extra-role contribution sends a strong signal that the organization values the relational and prosocial aspects of teaching, thereby reinforcing the social exchange cycle.

### Limitations and future research

This study has several limitations, which also indicate clear directions for future research. First, adopting a cross-sectional design, this study only reveals the correlational relationships among variables and cannot draw definitive causal inferences; future research should employ longitudinal or experimental designs to further verify the relational paths proposed in this study. Second, all data were collected via self-report measures, which may lead to common method bias. Future studies could adopt a multi-source assessment approach to address this issue, such as having school administrator's rate teachers' organizational citizenship behavior and colleagues assess the level of interpersonal trust. Third, this study employed convenience sampling to recruit participants, a choice driven by practical and strategic considerations including accessibility to participating schools, administrative cooperation, efficiency in large-scale data collection, and feasibility within the constraints of research time and resources. While this approach supported the successful implementation of the study, it may restrict the representativeness of the sample and limit the generalizability of the present findings to other teacher populations, regions, or educational contexts. Future studies are encouraged to use probability sampling or more geographically and demographically diverse sampling strategies to strengthen the external validity and broader applicability of results. Fourth, this study was conducted in the specific context of Chinese PreK-12 education, and the specificity of cultural and educational settings may restrict the cross-context applicability of the conclusions, thus replication studies in diverse cultural backgrounds and educational systems are urgently needed. Additionally, future research can further explore other mediating variables such as teacher self-efficacy and affective commitment to construct a more comprehensive mechanism model, and investigate potential moderating variables such as leadership styles (e.g., transformational leadership and authoritarian leadership) or school types (e.g., public and private schools) to clarify the boundary conditions of the research conclusions. In summary, this study identifies an important pathway through which psychological safety promotes Chinese PreK-12 teachers' organizational citizenship behavior, namely the mediating role of interpersonal trust cultivation. The findings indicate that investing in the social and emotional ecology of schools is not only an imperative in education settings but also a crucial strategic measure, and is of great significance for stimulating teachers' voluntary collective initiative and facilitating the high-quality development of education.

## Conclusions

This study explored the association between school organizational climate and teachers' proactive behaviors. The findings reveal that Chinese PreK-12 teachers' psychological safety is significantly positively correlated with their organizational citizenship behavior, with interpersonal trust playing a partial mediating role in this association. From the perspective of social exchange theory, teachers' perceived safe organizational climate shows a positive correlation with their level of interpersonal trust in colleagues and leaders, and such trust is closely associated with teachers' behaviors that go beyond formal job requirements, thus forming an associative pathway of “psychological safety—interpersonal trust—organizational citizenship behavior.” For the educational management practice of primary and secondary schools in China, these findings indicate that teachers' voluntary contributions are associated with both the cultivation of a safe school organizational climate and interpersonal trust. Attaching importance to the development of such psychological and social resources helps elicit teachers' positive behaviors that exceed their formal job responsibilities.

## Data Availability

The data analyzed in this study is subject to the following licenses/restrictions: the raw data supporting the conclusions of this article will be made available by the authors, without undue reservation. Requests to access these datasets should be directed to Baoan Feng, fba1986@126.com.
